# Exploring the role of sigma factor gene expression on production by *Corynebacterium glutamicum*: sigma factor H and FMN as example

**DOI:** 10.3389/fmicb.2015.00740

**Published:** 2015-07-22

**Authors:** Hironori Taniguchi, Volker F. Wendisch

**Affiliations:** Genetics of Prokaryotes, Faculty of Biology and Center for Biotechnology, Bielefeld UniversityBielefeld, Germany

**Keywords:** *Corynebacterium*, RNA polymerase sigma factor, *sigH*, ribA, riboflavin, FMN production

## Abstract

Bacteria are known to cope with environmental changes by using alternative sigma factors binding to RNA polymerase core enzyme. Sigma factor is one of the targets to modify transcription regulation in bacteria and to influence production capacities. In this study, the effect of overexpressing each annotated sigma factor gene in *Corynebacterium glutamicum* WT was assayed using an IPTG inducible plasmid system and different IPTG concentrations. It was revealed that growth was severely decreased when *sigD* or *sigH* were overexpressed with IPTG concentrations higher than 50 μM. Overexpression of *sigH* led to an obvious phenotypic change, a yellow-colored supernatant. High performance liquid chromatography analysis revealed that riboflavin was excreted to the medium when *sigH* was overexpressed and DNA microarray analysis confirmed increased expression of riboflavin biosynthesis genes. In addition, genes for enzymes related to the pentose phosphate pathway and for enzymes dependent on flavin mononucleotide (FMN), flavin adenine dinucleotide (FAD), or NADPH as cofactor were upregulated when *sigH* was overexpressed. To test if *sigH* overexpression can be exploited for production of riboflavin-derived FMN or FAD, the endogenous gene for bifunctional riboflavin kinase/FMN adenyltransferase was co-expressed with *sigH* from a plasmid. Balanced expression of *sigH* and *ribF* improved accumulation of riboflavin (19.8 ± 0.3 μM) and allowed for its conversion to FMN (33.1 ± 1.8 μM) in the supernatant. While a proof-of-concept was reached, conversion was not complete and titers were not high. This study revealed that inducible and gradable overexpression of sigma factor genes is an interesting approach to switch gene expression profiles and to discover untapped potential of bacteria for chemical production.

## Introduction

The sigma factor is a component of RNA polymerase holoenzyme and is important to recognize the promoter sequence in transcription initiation (Vassylyev et al., 2002; Feklístov et al., 2014). In general, a bacterium possesses two or more sigma factor genes and RNA polymerase holoenzymes with different sigma factors recognize distinct promoter sequences (Paget and Helmann, 2003; Staroń et al., 2009). Upon environmental stress the vegetative sigma factor may be replaced by an alternative sigma factor, a mechanism wide-spread in bacteria to cope with environmental changes (Kazmierczak et al., 2005; Sharma and Chatterji, 2010). This fundamental mechanism of transcriptional regulation has recently drawn attention as a candidate of metabolic engineering for global transcriptional engineering (Tripathi et al., 2014).

*Corynebacterium glutamicum* was isolated as a glutamate-producing organism in 1956 and has been used for the large scale production of glutamate and lysine for more than five decades (Eggeling and Bott, 2005, 2015; Burkovski, 2008; Yukawa and Inui, 2013). Amino acid producing strains have been developed based on random mutagenesis and/or rational engineering. For instance, this bacterium has been engineered to produce amino acids such as L-serine (Peters-Wendisch et al., 2005), L-isoleucine (Morbach et al., 1996), L-valine (Radmacher et al., 2002; Blombach et al., 2007), L-proline (Jensen and Wendisch, 2013), L-tryptophan (Ikeda and Katsumata, 1999), L-citrulline (Eberhardt et al., 2014), or L-arginine (Park et al., 2014). It has been also engineered to produce precursors of amino acids such as 2-ketoisovalerate (Krause et al., 2010) and 2-ketoisocaproate (Bückle-Vallant et al., 2014; Vogt et al., 2015) or amino acid-derived compounds such 1,4-diaminobutane (Schneider and Wendisch, 2010; Schneider et al., 2012) or 1,5-diaminopentane (Mimitsuka et al., 2007). Metabolic engineering focused mainly on amino acid biosynthesis, precursor supply, cofactor regeneration and amino acid transport. Concerning regulatory engineering, mainly feedback-resistant versions of key enzymes are in use, however, also transcriptional regulatory engineering has been applied, e.g., by deletion of the genes encoding pathway-specific regulators such as LbtR (Bückle-Vallant et al., 2014) or ArgR (Hwang et al., 2008) or higher order regulators such as SugR (Blombach et al., 2009). However, global regulatory engineering using sigma factor genes has not yet been explored.

*C. glutamicum* WT possesses seven sigma factor genes encoded on its chromosome (Kalinowski et al., 2003). These sigma factors are classified into group 1 (SigA), group 2 (SigB) and group 4 (SigC, SigD, SigE, SigH, SigM) according to their conserved structures. *C. glutamicum* lacks group 3 type sigma factors (Pátek and Nešvera, 2011). The regulons of some of these sigma factors have been studied, e.g., for SigA, SigB, SigE, SigH, and SigM. SigA is the principle sigma factor and related to the transcription initiation of housekeeping genes (Pfeifer-Sancar et al., 2013). The gene *sigA* is essential in *C. glutamicum* as well as in other bacteria (Pátek and Nešvera, 2011). SigB is related to the general stress response and assumed to play an important role at the transition from the exponential to the stationary growth phase (Larisch et al., 2007). Analysis of the *sigB* deletion mutant revealed that SigB is involved in glucose metabolism under oxygen deprivation conditions, thymidylate synthesis and protein secretion (Ehira et al., 2008; Cho et al., 2012; Watanabe et al., 2013). The functions of SigC and SigD have not yet been elucidated. SigE is related to surface stress and its activity is repressed by anti-sigma factor CseE (Park et al., 2008). SigH is involved in the response to heat shock, pH stress and disulfide/oxidative stress (Kim et al., 2005a; Ehira et al., 2008; Barriuso-Iglesias et al., 2013), and its activity is repressed by anti-sigma factor RshA (Busche et al., 2012). Recently, SigH-dependent promoters were studied by ChIP-chip analysis (Toyoda et al., 2015). SigM is involved in transcription of disulfide stress related genes (Nakunst et al., 2007).

In this study, the effects of graded sigma factor gene overexpression on *C. glutamicum* have been characterized. Based on the finding that *sigH* overexpression resulted in riboflavin production, flavin mononucleotide (FMN) producing *C. glutamicum* strains have been constructed.

## Materials and Methods

### Bacterial Strains, Plasmid, and Primer

The strains, plasmids and oligonucleotides used in this work are listed in **Table 1**. Plasmids were constructed based on pEKEx3 and pVWEx1, IPTG inducible *Escherichia coli* – *C. glutamicum* shuttle vectors (Peters-Wendisch et al., 2001; Stansen et al., 2005). The DNA sequence of sigma factor gene was amplified from genomic DNA of *C. glutamicum* WT by polymerase chain reaction (KOD, Novagen, Darmstadt, Germany) with respective primer pairs in **Table 1**. The PCR product was inserted into BamHI-digested pEKEx3 or pVWEx1 plasmid by Gibson assembly (Gibson et al., 2009). *E. coli* DH5α was used for cloning. *E. coli* competent cells were transformed by heat shock method (Sambrook, 2001) or by electroporation method (Nováková et al., 2014). All cloned DNA fragments were confirmed to be correct by sequencing. *C. glutamicum* competent cells were transformed by electroporation at 2.5 kV, 200 Ω, and 25 μF (van der Rest et al., 1999; Eggeling and Bott, 2005).

**Table 1:** Bacterial strains, plasmids and oligonucleotides used in this study.

Bacterial strain	Relevant characteristic	Reference
*Escherchia coli*		
DH5α	F-*thi*-1 *endA*1 *hsdR*17(r-, m-) *supE*44 Δ*lacU*169 (Φ80*lacZ*ΔM15) *recA*1 *gyrA*96 *relA*1	Bethesda Research Laboratories
*Corynebacterium glutamicum*		
WT	Wild type, ATCC 13032	ATCC

**Plasmid**	**Relevant characteristic**	**Reference**

pEKEx3	SpecR; *E. coli*–*C. glutamicum* shuttle vector for regulated gene expression (Ptac, *lacI*q, pBL1 oriVCg)	Stansen et al. (2005)
pVWEx1	KanR; *E. coli*–*C. glutamicum* shuttle vector for regulated gene expression (Ptac, *lacI*q, pCG1 oriVCg)	Peters-Wendisch et al. (2001)
pEKEx3-*sigA*	SpecR, pEKEx3 with *sigA* from *C. glutamicum* WT	This study
pEKEx3-*sigB*	SpecR, pEKEx3 with *sigB* from *C. glutamicum* WT	This study
pEKEx3-*sigC*	SpecR, pEKEx3 with *sigC* from *C. glutamicum* WT	This study
pEKEx3-*sigD*	SpecR, pEKEx3 with *sigD* from *C. glutamicum* WT	This study
pEKEx3-*sigE*	SpecR, pEKEx3 with *sigE* from *C. glutamicum* WT	This study
pEKEx3-*sigH*	SpecR, pEKEx3 with *sigH* from *C. glutamicum* WT	This study
pEKEx3-*sigM*	SpecR, pEKEx3 with *sigM* from *C. glutamicum* WT	This study
pVWEx1-*sigH*	KanR, pVWEx1 with *sigH* from *C. glutamicum* WT	This study
pEKEx3-*ribF*	SpecR, pEKEx3 with *ribF* from *C. glutamicum* WT	This study
pVWEx1-*ribF*	KanR, pVWEx1 with *ribF* from *C. glutamicum* WT	This study

**Oligonucleotide**	**Sequence (5′-3′)**	**Reference**

*sigA*-fwd	GCCTGCAGGTCGACTCTAGAG***GAAAGGAGG***CCCTTCAG**ATG**GTAGAAAACAACGTAGCAAAAAAGACGGTCG	This study
*sigA*-rev	CGGTACCCGGGGATCTTAGTCCAGGTAGTCGCGAAGGACCTG	This study
*sigB*-fwd	GCCTGCAGGTCGACTCTAGAG***GAAAGGAGG***CCCTTCAG**ATG**ACAGCACCGTCCACGCAG	This study
*sigB*-rev	CGGTACCCGGGGATCTTACTGGGCGTACTCACGAAGACGTG	This study
*sigC*-fwd	GCCTGCAGGTCGACTCTAGAG***GAAAGGAGG***CCCTTCAG**GTG**AAGTCAAAAGAGCGTAACGACGC	This study
*sigC*-rev	CGGTACCCGGGGATCCTAACCTTGGGCGGATTTGCCATCTTCG	This study
*sigD*-fwd	GCCTGCAGGTCGACTCTAGAG***GAAAGGAGG***CCCTTCAG**TTG**GCTGATACTGAGCGCGAGCTC	This study
*sigD*-rev	CGGTACCCGGGGATCTTACTTGTTCTCCTGCTGCTCAAGTGTGCTTC	This study
*sigE*-fwd	GCCTGCAGGTCGACTCTAGAG***GAAAGGAGG***CCCTTCAG**ATG**ACTTATATGAAAAAGAAGTCCCGAGATGACGCAC	This study
*sigE*-rev	CGGTACCCGGGGATCTTAGTGGGTTGGAACCAACAAAGAAACTTCCTCG	This study
*sigH*-fwd	GCCTGCAGGTCGACTCTAGAG***GAAAGGAGG***CCCTTCAG**ATG**GCTGAAAACCGAACCGGCAC	This study
*sigH*-rev	CGGTACCCGGGGATCTTATGCCTCCGAATTTTTCTTCATGTCGGGATG	This study
*sigM*-fwd	GCCTGCAGGTCGACTCTAGAG***GAAAGGAGG***CCCTTCAG**ATG**ACAGTACTGCCTAAAAACCATGACCTAAGC	This study
*sigM*-rev	CGGTACCCGGGGATCTCAGTTGCTTTCGCACTGTATGGAGCC	This study
*ribF*-fwd	GCCTGCAGGTCGACTCTAGAG***GAAAGGAGG***CCCTTCAG**GTG**GATATTTGGAGTGGACT	This study
*ribF*-rev	CGGTACCCGGGGATCTTAAGCGCTGGGCTGGGTGT	This study

*Underlined sequences represent the overlap region with vector plasmid; sequences in bold italic represent ribosome binding sites; sequences in bold represents the translational start codons.*

### Medium and Growth Condition

*C. glutamicum* was precultured in BHI or LB medium overnight, washed once with CGXII medium (Eggeling and Bott, 2005) without carbon source and inoculated in CGXII with 222 mM of glucose at initial OD at 600 nm of 1. The OD was measured with UV-1202 spectrophotometer (Shimadzu, Duisburg, Germany) with suitable dilutions. When appropriate, 100 μg/mL of spectinomycin, 25 μg/mL of kanamycin and IPTG were added. Growth experiment with Biolector^®^ cultivation system (m2pLabs, Baesweiler, Germany) was performed in 1 mL of CGXII using FlowerPlate^®^ (m2pLabs, Baesweiler, Germany) at 30°C, 1,100 rpm. Cell growth was monitored online every 10 min for 48 h. Maximum growth rate μ (h^-1^) was calculated from 20 measuring points of arbitrary unit of backscattering light (620 nm). Plate image was scanned with Perfection V750-M Pro scanner (Epson, Ludwigshafen am Rhein, Germany). Color balance of blue against yellow was set to +70.

### Riboflavin Production Experiments

Riboflavin production experiments were performed at 30°C, 120 rpm in 50 mL of GCXII with 222 mM of glucose and 15 μM of IPTG using 500 mL baﬄed flasks. Supernatant was separated by centrifugation after 48 h of cultivation. Riboflavin concentration of cell-free supernatant was analyzed using high performance liquid chromatography (HPLC; Agilent Technologies Sales & Services GmbH & Co. KG, Waldbronn, Germany). The confirmation and quantification of riboflavin was performed using diode array detector (DAD). Samples were separated with a column system consisting of a precolumn (LiChrospher 100 RP18 EC-5μ (40 mm × 4 mm), CS-Chromatographie Service GmbH, Langerwehe, Germany) and a main column (LiChrospher 100 RP18 EC-5μ (125 mm × 4 mm), CS Chromatographie Service GmbH, Langerwehe, Germany) with 0.1 M sodium acetate, pH 7.2 supplemented with 0.03% sodium azide (A) and methanol (B) as the mobile phase. The following gradient was used at a flow rate of 1.2 mL/min; 0 min B: 20%, 0.5 min B: 38%, 2.5 min B: 46%, 3.7 min B: 65%, 5.5 min B: 70%, 6 min B: 75%, 6.2 min B: 85%, 6.7 min B: 20%, 8.9 min B: 20%.

### Transcriptome Analysis of *sigH* Overexpressing Strain using DNA Microarrays

*C. glutamicum* strains WT(pEKEx3) and WT(pEKEx3-*sigH*) were cultured in BHI medium and inoculated into CGXII medium with 222 mM of glucose for adaptation. Cells were cultured overnight and inoculated into 50 mL of CGXII medium with 222 mM of glucose and 10 or 15 μM of IPTG at the initial OD of 1. Cells were harvested in the early exponential growth phase (OD between 6 and 8) and RNA isolation was performed as described previously (Wendisch, 2003). The purified RNA was analyzed by spectrophotometer (NanoDrop) for quantity and gel electrophoresis for quality. The RNA sample was stored at -80°C until further use. cDNA synthesis from total RNA as well as DNA microarray hybridization were performed as described previously (Netzer et al., 2004; Polen et al., 2007). Normalization and evaluation of the microarray data was done with the software package EMMA 2 (Dondrup et al., 2009). Genes which were upregulated in WT(pEKEx3-*sigH*) under both 10 and 15 μM of IPTG concentration were taken into account for further analysis (*p*-value < 0.05, *M*-value > 1).

### Measurement of Glucose-6-Phosphate 1-Dehydrogenase Enzyme Activities

Enzyme activities of glucose-6-phosphate 1-dehydrogenase in *C. glutamicum* WT (pEKEx3) and *C. glutamicum* WT(pEKEx3-*sigH*) were measured in cell free crude extracts, which were prepared as described previously (Stansen et al., 2005) with some modification. Shortly, cells grown in CGXII medium with 222 mM of glucose and 15 μM of IPTG were harvested in the exponential growth phase (OD around 6), washed once with disruption buffer (50 mM Tris-HCl pH 8.5, 10 mM MgCl_2_, and 1 mM DTT) and stored at -20°C until use. Protein concentrations were determined with the Bradford reagent using bovin serum albumin as a standard. Enzyme activities were measured spectrophotometrically following NADPH formation at 30°C in final volume of 1 mL. The concomitant formation of NADPH was measured at 340 nm and absorption coefficient of 6.3 mM^-1^ cm^-1^ at 340 nm was used for calculating enzyme activities. The assay contained 50 mM Tris-HCl pH 8.5, 10 mM MgCl_2_, 100 mM NADP^+^ and 100 mM glucose-6-phosphate.

### FMN and FAD Production Experiments

FMN and FAD production experiment was performed at 30°C with 120 rpm in 50 mL of GCXII with 222 mM of glucose using 500 mL baﬄed flasks. 100 μM of IPTG was added after OD reached around 10. Supernatant was separated by centrifugation after 48 h of cultivation. FMN and FAD concentration of cell-free supernatant was analyzed as described previously with some modifications (Barile et al., 1997). Shortly, signal was detected with fluorescent detector (FLD; excitation and emission wavelengths of 450 and 520 nm, respectively) and samples were separated with the same column systems used in riboflavin production experiments with 20 mM potassium phosphate, pH 6.0 (A) and methanol (B) as the mobile phase. The following ratio was used at a flow rate of 1.0 mL/min; 0–5 min B: 25%, 5–10 min B: 50%.

## Results

### Effect of Overexpressing Sigma Factor Genes in *C. glutamicum*

**FIGURE 1:**
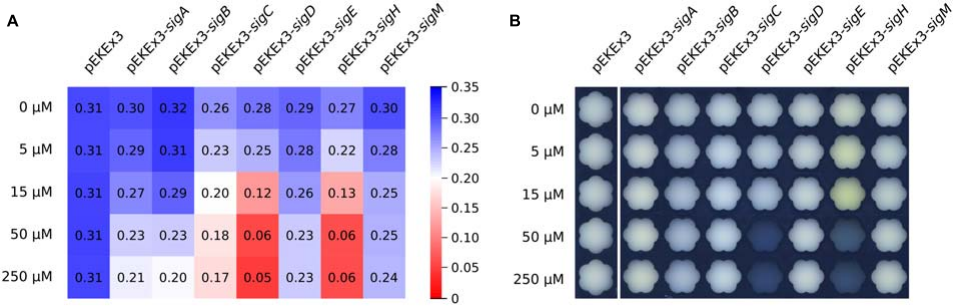
**Maximum growth rates of cultivations of *C. glutamicum* wild type (WT) transformed with plasmids for overexpression of various sigma factor genes (A) and images of these cultures taken after 48 h of cultivation (B)**. CGXII with 222 mM of glucose was used as growth medium. The different IPTG concentrations used to induce sigma factor gene overexpression are indicated. The growth rates (h^-1^) depicted in **(A)** are color-coded from red to blue according to the given scale.

**FIGURE 2:**
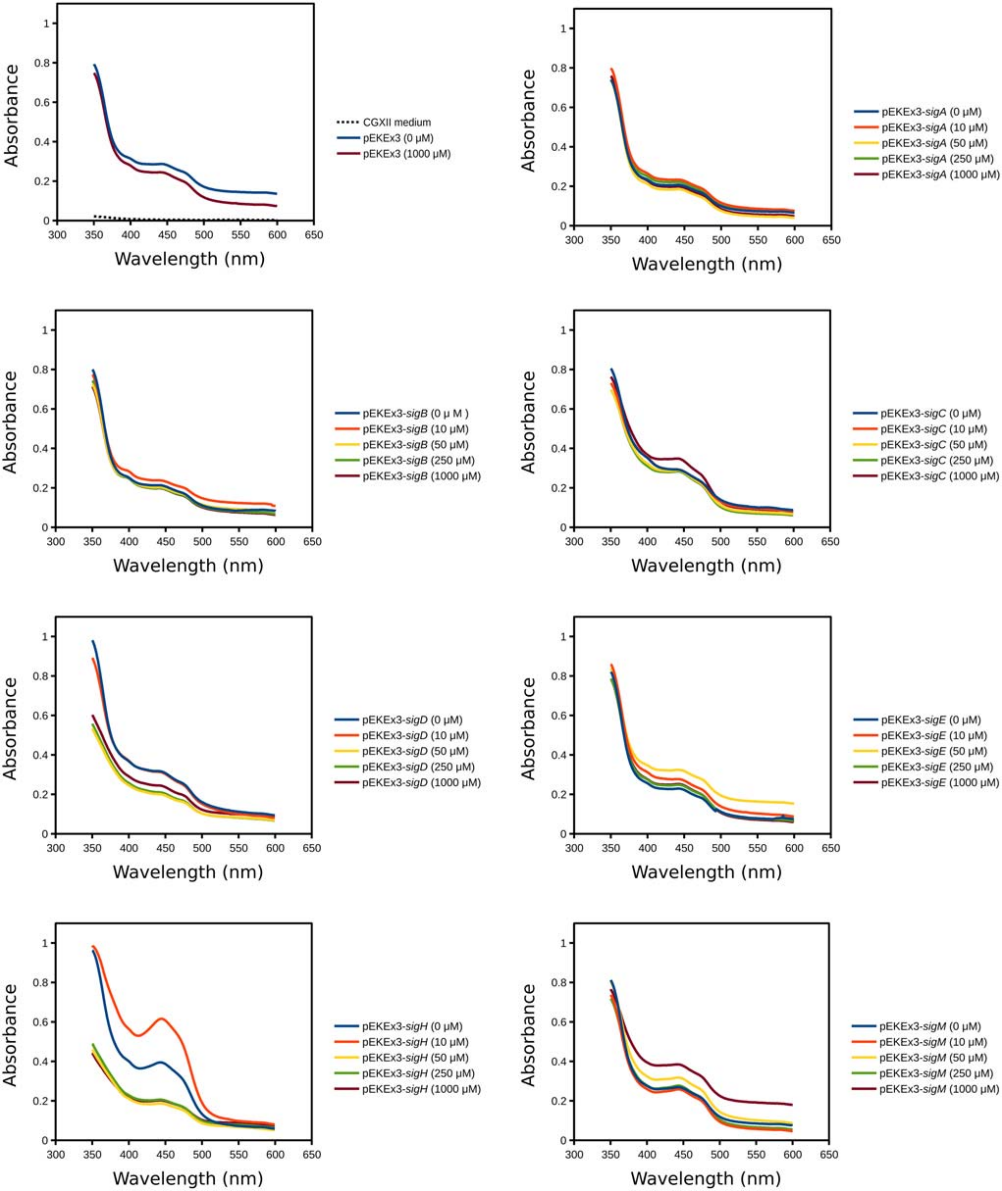
**Absorption spectra of supernatants of cultures of *C. glutamicum* WT transformed with plasmids for overexpression of various sigma factor genes.** CGXII with 222 mM of glucose was used as growth medium. The IPTG concentrations and the plasmids used for overexpression of sigma factor genes are indicated. Supernatants were analysed after 48 h of cultivation.

To investigate the influence of overexpressing sigma factor genes in *C. glutamicum*, each sigma factor gene (*sigA*, *sigB*, *sigC*, *sigD*, *sigE*, *sigH*, and *sigM*) was cloned into IPTG-inducible expression vector pEKEx3 and transformed into *C. glutamicum* WT. Growth of these strains and of a control strain containing the empty vector pEKEx3 was monitored in the presence of different IPTG concentrations (0, 5, 15, 50, or 250 μM) in CGXII medium containing 222 mM of glucose. Growth of the control strains was not affected by IPTG, while sigma factor gene transformants grew with lower growth rates at higher IPTG concentrations. In particular, *sigD* and *sigH* transformants exhibited strongly reduced growth rates with 50 and 250 μM IPTG and did not reach the stationary phase during 48 h of cultivation (**Figure 1A**). Interestingly, the cultures of the *sigH* transformant with up to 15 μM IPTG were colored yellow (**Figure 1B**). Therefore, the supernatants of all cultures were analyzed by recording absorbance spectra from 350 to 600 nm (**Figure 2**). While absorbance of the different supernatants varied to some degree when comparing the different transformants, the supernatant of *sigH* transformant induced with 10 μM IPTG showed a strong absorbance centered at about 450 nm. Since the *sigH* transformant did not grow when induced with higher IPTG concentrations, this absorbance peak was not observed under these conditions.

### Overexpression of *sigH* Resulted in Riboflavin Secretion

**FIGURE 3:**
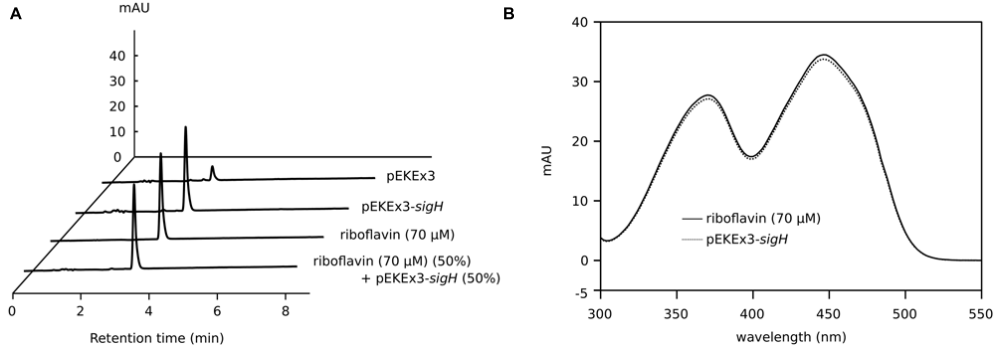
**Analysis of supernatants of *C. glutamicum* WT(pEKEx3-*sigH*) cultures by **(A)** high performance liquid chromatography (HPLC) and **(B)** spectrophotometry. (A)** HPLC chromatograms of supernatants of *C. glutamicum* WT(pEKEx3) and WT(pEKEx3-*sigH*) after 48 h in CGXII with 222 mM of glucose. Expression of *sigH* was induced by addition of 15 μM of IPTG at the start of the cultivation. A standard of pure riboflavin (70 μM) and a 50%/50% mixture of this standard and the supernatant of the culture of WT(pEKEx3-*sigH*) are given for comparison. Absorbance at 450 nm is shown. **(B)** Spectra recorded at the retention time of 3.2 min of the HPLC samples of *C. glutamicum* WT(pEKEx3-*sigH*) and the riboflavin standard from **(A)**.

**FIGURE 4:**
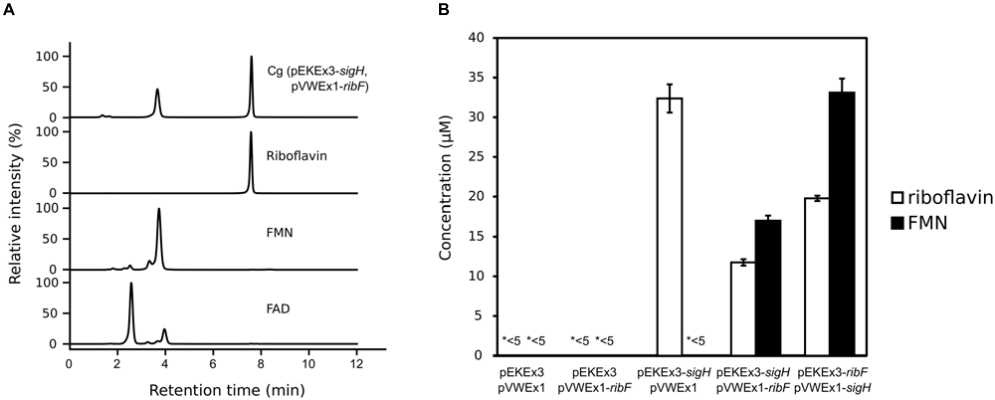
**Analysis of supernatants of *C. glutamicum* WT(pEKEx3-*sigH*, pVWEx1-*ribF*) cultures by HPLC **(A)** and riboflavin and FMN concentrations in supernatants of various strains (B). (A)** HPLC chromatograms of supernatants of *C. glutamicum* WT(pEKEx3-*sigH*, pVWEx1-*ribF*) after 48 h in CGXII with 222 mM of glucose. Expression of *sigH* was induced by addition of 100 μM of IPTG in the middle of the exponential growth phase (OD ∼10). Standards of commercial preparations of riboflavin, FMN and FAD are given for comparison. Absorbance at 450 nm is shown. **(B)** Concentrations of riboflavin and FMN in supernatants of cultures of *C. glutamicum* WT transformed with the indicated plasmids. FAD was not detectable (<5 μM) in the analyzed supernatants. *<5 indicates that riboflavin or FMN in these supernatants were below 5 μM. Biological triplicates.

To verify the yellow color phenotype of WT(pEKEx3-*sigH*) in a different cultivation setting, this strain was grown in shake flasks and induced with 15 μM IPTG immediately after inoculation. The cultures in shake flasks and the supernatants showed yellow color. Spectrophotometric analysis of the supernatant from the culture of WT(pEKEx3-*sigH*) revealed maximal absorption at 450 nm as well as yellow fluorescence under UV irradiation (data not shown). Since the spectral properties of riboflavin fit well to those observed here, the supernatant and riboflavin as standard were analyzed by HPLC. Co-elution at around 3.2 min of riboflavin with the compound in the supernatant of WT(pEKEx3-*sigH*; **Figure 3A**) and comparable absorption spectra (300–550 nm; **Figure 3B**) revealed that riboflavin was produced by *C. glutamicum* WT(pEKEx3-*sigH*). No other significant peak was detected. Quantification based on a series of suitable riboflavin concentrations indicated that the accumulation of riboflavin in the supernatant of WT(pEKEx3-*sigH*) was about seven times as high as that of control strain WT(pEKEx3), (68.0 ± 1.3 μM and 10.4 ± 1.5 μM, respectively, biological triplicates). When expression of *sigH* was induced by addition of 100 μM of IPTG in the middle of the exponential growth phase (OD ∼10) about 35 μM riboflavin accumulated (**Figure 4**).

### Global Gene Expression Changes due to *sigH* Overexpression

To determine if *sigH* overexpression affects riboflavin biosynthesis genes, DNA microarray experiments with *C. glutamicum* WT(pEKEx3-*sigH*) were performed and global gene expression at two different IPTG concentrations (10 and 15 μM) was compared to the control strain WT(pEKEx3). Statistically significant gene expression increases of at least two fold were observed for 193 and 142 genes, respectively, upon induction with 10 and 15 μM of IPTG (*M*-value > 1, *p*-value < 0.05; **Table 2**). Fifty genes were considered further as they were upregulated in both IPTG concentrations. Among these, genes related to riboflavin synthesis [*ribH* (cg1797), *ribA* (cg1798), *ribC* (cg1799)] and the pentose phosphate pathway [*zwf* (cg1778), *opcA* (cg1779)] were found. In addition, many genes encoding NADPH-dependent or FAD/FMN-dependent oxidoreductases were upregulated upon *sigH* overexpression (**Table 2**)

**Table 2:** DNA microarray analysis of genes differentially expressed upon *sigH* overexpression.

			*M*-value^b^		*P*-value^c^	
Gene ID^a^	Gene name^a^	Function of protein^a^	10 μM	15 μM	10 μM	15 μM
cg0184		Conserved hypothetical protein	1.9	1.2	1.6E-2	2.6E-2
cg0186		Putative methylated-DNA-protein-cysteine methyltransferase	1.3	1.1	4.3E-4	3.8E-3
cg0614		Hypothetical protein	2.9	2.2	2.1E-3	5.6E-3
cg0616	*fdhD*	Putative formate dehydrogenase, FdhD-family	2.7	3.4	4.8E-4	1.1E-2
cg0617		Hypothetical protein	2.1	2.6	3.2E-4	8.8E-4
cg0876	*sigH*	RNA polymerase sigma factor, ECF-family	4.1	4.4	1.4E-5	6.0E-4
cg1081		ABC-type putative daunorubicin transporter, ATPase subunit	1.2	1.4	1.6E-2	2.9E-2
cg1127		Putative mycothiol *S*-conjugate amidase	1.3	2.6	1.3E-3	2.7E-3
cg1386	*fixA*	Putative electron transfer flavoprotein, beta subunit	1.1	2.0	1.7E-3	1.4E-2
cg1397	*trmU*	tRNA (5-methylaminomethyl-2-thiouridylate)-methyltransferase	1.5	1.7	6.6E-4	4.8E-3
cg1398		Conserved hypothetical protein	1.7	2.4	1.9E-2	2.3E-2
cg1432	*ilvD*	Dihydroxy-acid dehydratase	1.9	1.9	2.0E-4	6.7E-4
cg1628		Putative hydrolase, alpha/beta superfamily	2.5	1.9	4.8E-2	9.1E-3
cg1671		Putative membrane-associated GTPase	1.7	1.3	3.9E-2	1.4E-2
cg1687		Putative transcriptional regulatory protein	1.4	1.3	1.9E-2	9.7E-3
cg1688		Putative proteasome component	2.2	2.2	2.0E-4	7.9E-3
cg1689		Conserved hypothetical protein	2.3	3.0	9.5E-4	1.4E-2
cg1709	*mshC*	Putative 1-D-myo-inosityl-2-amino-2-deoxy-alpha-D-glucopyranoside-L-cysteine ligase	2.9	1.9	1.2E-4	1.5E-3
cg1764	*sufB*	FeS assembly membrane protein, SufB-family	1.2	1.0	1.8E-3	9.1E-3
cg1776	*tal*	Transaldolase	1.0	1.7	1.6E-2	1.2E-3
cg1778	*zwf*	Glucose-6-phosphate 1-dehydrogenase	1.2	2.0	4.1E-3	4.6E-3
cg1779	*opcA*	Glucose-6-phosphate 1-dehydrogenase subunit	1.6	1.2	1.5E-3	5.0E-2
cg1796	*ribX*	Conserved putative membrane protein, RibX-like	1.2	1.7	6.7E-3	3.5E-3
cg1797	*ribH*	Riboflavin synthase, beta chain	1.7	1.3	6.4E-5	1.7E-3
cg1798	*ribA*	Putative GTP cyclohydrolase II/3,4-dihydroxy-2-butanone-4-phosphatesynthase	2.0	1.0	4.6E-5	8.0E-3
cg1799	*ribC*	Riboflavin synthase, alpha chain	1.9	3.0	8.8E-4	2.7E-2
cg2078		Peptide methionine sulfoxide reductase	3.3	2.8	4.4E-5	4.2E-5
cg2079		Conserved hypothetical protein	1.4	1.1	2.2E-3	7.7E-3
cg2106		Conserved hypothetical protein	2.7	5.0	3.1E-3	4.5E-3
cg2127		Hypothetical protein	1.1	1.8	8.7E-3	3.5E-2
cg2194	*mtr*	Putative NADPH-dependent mycothiol reductase	3.1	2.9	2.9E-8	2.2E-3
cg2206	*ispG*	4-hydroxy-3-methylbut-2-en-1-yl diphosphate synthase	1.3	1.2	7.5E-3	2.7E-2
cg2247		Hypothetical protein	1.8	2.0	2.4E-5	3.1E-4
cg2296	*hisI*	Phosphoribosyl-AMP cyclohydrolase	1.2	1.1	3.1E-3	8.4E-3
cg2297	*hisF*	Imidazole glycerol phosphate synthase subunit HisF	1.4	1.4	1.6E-2	9.7E-4
cg2411		Conserved hypothetical protein, HesB/YadR/YfhF family	2.1	2.8	4.0E-4	4.2E-4
cg2423	*lipA*	Lipoyl synthetase	1.6	1.7	1.9E-4	8.5E-3
cg2538		Alkanal monooxygenase (FMN-linked)	3.3	4.2	7.9E-4	1.6E-5
cg2644	*clpP2*	Endopeptidase Clp, proteolytic subunit	1.1	1.1	7.6E-4	9.4E-5
cg2661		Putative dithiol-disulfide isomerase	1.5	1.9	1.1E-4	4.7E-3
cg2665		Hypothetical protein	1.4	1.4	4.6E-3	7.8E-3
cg2762	*murI*	Glutamate racemase	2.0	2.4	5.0E-2	2.4E-2
cg2835		Putative acetyltransferase	1.0	3.3	4.7E-2	2.6E-2
cg2838		Putative dithiol-disulfide isomerase	3.6	3.2	3.9E-6	2.9E-3
cg3236	*msrA*	Protein-methionine-*S*-oxide reductase	1.4	3.4	3.4E-3	1.1E-2
cg3372		Conserved hypothetical protein	1.1	1.3	2.5E-5	4.2E-2
cg3405		NADPH:quinone reductase Zn-dependent oxidoreductase	2.6	2.8	7.8E-4	1.7E-2
cg3422	*trxB*	Thioredoxin reductase	1.8	2.2	3.1E-4	1.8E-4
cg3423	*trxC*	Thioredoxin	1.5	2.1	4.1E-4	6.8E-5
cg3424	*cwlM*	*N*-acetylmuramoyl-L-alanine amidase	1.3	1.7	8.2E-3	1.4E-2

*^a^Gene ID, gene name and function of proteins are given according to CoryneRegNet (http://coryneregnet.de/). ^b^Relative RNA levels in a strain overexpressing *sigH* as compared to the empty vector control are shown as log 2 values (*M*-values). To induce sigH overexpression either 10 or 15 μM IPTG were added. ^c^*P*-values were determined by Student’s *t*-test.*

To confirm the observed gene expression changes of *zwf* and *opcA*, the specific enzyme activity of glucose-6-phosphate dehydrogenase encoded by *zwf* and *opcA* was measured. The specific activity of glucose-6-phosphate dehydrogenase in the crude extracts of *C. glutamicum* WT(pEKEx3-*sigH*) was three times as high as in those of *C. glutamicum* WT(pEKEx3) (117 ± 7 and 35 ± 4 mU/mg, respectively, biological triplicates). Thus, *sigH* overexpression led to increased *zwf* and *opcA* mRNA level and increased specific activity of the encoded glucose-6-phosphate dehydrogenase.

### FMN Production by *C. glutamicum* Established as Proof-of-Concept based on Overexpression of Endogenous Genes *sigH* and *ribF*

Riboflavin is the precursor of FMN (flavin mononucleotide) and FAD (flavin adenine dinucleotide), which are biologically important as redox cofactor for many flavoenzymes and have an advantage as food additives over riboflavin due to much higher solubility in water (Kirk-Othmer, 1984). *C. glutamicum* possesses one gene, *ribF* (cg2169), encoding putative bifunctional riboflavin kinase / FMN adenylyltransferase, which converts riboflavin to FMN and FAD. In *C. glutamicum*, *ribF* is located about 350 kb downstream of the *def2-fmt-fmu-rpe-ribGACH* operon that contains the riboflavin biosynthesis genes *ribG*, *ribA*, *ribC*, and *ribH*. Since the *ribF* mRNA level was not affected notably by *sigH* overexpression (**Table 2**), simultaneous overexpression of *ribF* and *sigH* was tested. However, severely retarded growth was observed already with only 15 μM IPTG (data not shown). Therefore, expression of *ribF* or/and *sigH* was induced in the middle of the exponential growth phase (OD ∼10) using two compatible IPTG inducible plasmids, pEKEx3 and pVWEx1, with 100 μM of IPTG. After 48 h, neither riboflavin, FMN nor FAD were detected (<5 μM) in the supernatants of the control strain carrying the empty vectors. Expression of only *ribF* from pVWEx1 did not result in accumulation of riboflavin, FMN nor FAD. When only *sigH* was overexpressed from plasmid pEKEx3 riboflavin was secreted to the medium (32.4 ± 1.8 μM), but neither FMN nor FAD accumulated (**Figure 4**). However, when both genes were overexpressed in *C. glutamicum* WT(pEKEx3-*sigH*, pVWEx1-*ribF*) secretion of FMN (17.0 ± 0.6 μM) in addition to riboflavin (11.8 ± 0.4 μM) was detected, while FAD was not detected (<5 μM). To test if a different gene dosage affects FMN production, *sigH* was expressed from low copy number plasmid pVWEx1 and *ribF* from medium copy number plasmid pEKEx3. *C. glutamicum* WT(pEKEx3-*ribF*, pVWEx1-*sigH*) accumulated about two times higher concentrations of riboflavin (19.8 ± 0.3 μM) and FMN (33.1 ± 1.8 μM).

## Discussion

In this study the potential of overexpressing sigma factor genes for metabolic engineering of *C. glutamicum* was tested. Sigma factors are related to the promoter selectivity during transcription initiation and are expected to affect expression of larger groups of genes, e.g., RpoS of *E. coli* regulates 481 genes under different growth and stress conditions (Weber et al., 2005). However, there are examples of sigma factors relevant for expression of only few genes, e.g., FecI of *E. coli* that is involved in expression of only seven genes (Cho et al., 2014). The functions of the seven sigma factors of *C. glutamicum*, which for comparison possesses 127 DNA-binding transcriptional regulators (Brune et al., 2005), have not been studied in detail although SigB, SigE, SigH, and SigM have been studied by several groups (Kim et al., 2005a; Larisch et al., 2007; Nakunst et al., 2007; Ehira et al., 2008, 2009; Park et al., 2008; Pátek and Nešvera, 2011; Busche et al., 2012; Holátko et al., 2012; Toyoda et al., 2015). Here, we have determined the growth response of *C. glutamicum* to sigma factor gene overexpression. Overexpression of every sigma factor gene slowed growth in glucose minimal medium, however, the effects varied. The smallest effects were found when the general sigma factor genes *sigA* and *sigB* or the genes for SigE and SigM were overexpressed (**Figure 1**). Overexpression of *sigC* in glucose minimal medium with 250 μM IPTG reduced the growth rate by about one third (**Figure 1**). The growth was severely inhibited (about half-maximal inhibition with IPTG concentrations as low as 15 μM) as consequence of overexpressing *sigD* or *sigH*. The functions and promoter selectivities of SigC and SigD remain to be studied, however, it is known that deletion of *sigD* retarded growth under microaerobic conditions (Ikeda et al., 2009). The observed growth inhibitory effects of overexpressing *sigD* or *sigC* described in this study suggested that these sigma factors are not negligible and proper expression levels of those sigma factors are important for expression of genes required for fast growth in glucose minimal medium.

Analysis of the supernatants of *C. glutamicum* overexpressing sigma factor genes (**Figure 2**) revealed that only *sigH* overexpression led to the production of a colored compound, which was identified to be riboflavin (**Figure 3**). Moreover, *sigH* overexpression slowed growth (**Figure 1**). Regulation by SigH in *C. glutamicum* is known to some detail. The alternative sigma factor SigH is controlled by anti-sigma factor RshA, which possibly shuts down the SigH-dependent stress response after the cells have overcome the stress condition (Busche et al., 2012). SigH has been shown to be involved in expression of *trxB* encoding thioredoxin reductase (Kim et al., 2005a), *whcE* encoding transcriptional regulator WhiB (Kim et al., 2005b), *sigM* (Nakunst et al., 2007), small antisense RNA gene *arnA* (Zemanová et al., 2008), the F0F1-ATP synthase operon *atpBEFHAGDC* (Barriuso-Iglesias et al., 2013), mycothiol peroxidase gene *mpx* (Si et al., 2015a), mycothiol S-conjugate amidase gene *mca* (Si et al., 2014), and methionine sulfoxide reductase A gene *msrA* (Si et al., 2015b). In addition, promoter selectivity of SigH has been studied using an *in vitro* transcription system (Holátko et al., 2012). Moreover, the SigH regulon has been studied by DNA microarray and ChIP-chip analyses involving deletion and overexpression of *sigH* as well as deletion of the anti-sigma factor gene *rshA* (Ehira et al., 2009; Busche et al., 2012; Toyoda et al., 2015). The strong growth inhibition as a result of overexpression of *sigH* shown here is commensurate with the described functions of SigH. In our DNA microarray analysis, 50 genes were upregulated when *sigH* overexpression was induced with 10 and 15 μM IPTG (**Table 2**). These data generally agree with previous data on control by SigH (Ehira et al., 2009; Busche et al., 2012; Toyoda et al., 2015). Notably, overexpression of *sigH* in the wild type, i.e., in the presence of its anti-sigma factor RshA, elicited similar expression changes as deletion of *rshA*, i.e., 43 out of 50 genes upregulated as consequence of *sigH* overexpression were also upregulated in the absence of anti-sigma factor RshA (Busche et al., 2012). A motif search with the 50 upregulated genes (**Table 2**) using UniProt database (http://www.uniprot.org/) identified putative iron sulfur cluster-containing proteins encoded by cg0616 (*fdhD*), cg1432 (*ilvD*), cg2206 (*ispG*) and cg2423 (*lipA*), proteins predicted to contain NAD(P)H binding sites encoded by cg0184, cg0616 (*fdhD*), cg1778 (*zwf*), cg2194 (*mtr*), and cg3405, and proteins with predicted FMN/FAD binding sites encoded by cg0616 (*fdhD*), cg1386 (*fixA*), cg2194 (*mtr*), cg2538 and cg3422 (*trxB*). Iron sulfur clusters are sensitive to oxidative stress and NAD(P)H, FMN, FAD are important electron donor/acceptors. Upregulation of genes related to riboflavin synthesis under *sigH* overexpression observed here (**Table 2**) was consistent with a very recent ChIP-chip data on SigH-dependent promoters in *C. glutamicum* R (Toyoda et al., 2015).

In *C. glutamicum*, riboflavin biosynthesis was shown to be dependent on *ribA*-encoded bifunctional GTP cyclohydrolase II/3,4-dihydroxy-2-butanone 4-phosphate synthase, since in its absence efficient growth required supplemental riboflavin (Takemoto et al., 2014). Uptake of supplemental riboflavin occurs via the transporter RibM (Vogl et al., 2007) and both RibM protein levels and *ribM* mRNA were reduced in FMN-rich cells due to the FMN-riboswitch (Takemoto et al., 2014). The FMN-riboswitch has been observed in an RNAseq-based analysis of the transcriptional landscape of *C. glutamicum* (Pfeifer-Sancar et al., 2013) and control by the FMN-riboswitch was shown to involve Rho and RNase E/G (Takemoto et al., 2015). However, riboflavin biosynthesis appears not to be controlled by the FMN-riboswitch. Instead, transcription of the riboflavin biosynthesis operon depends on SigH and deletion of *rshA* and overexpression of *sigH* resulted in riboflavin secretion as recently reported in the *rshA* deletion mutant (**Figure 3**; Toyoda et al., 2015). Neither FMN nor FAD accumulated under these conditions, which may be explained by the fact that *ribF* expression has not been found to be influenced by deletion of *rshA* and overexpression of *sigH* (Busche et al., 2012; Toyoda et al., 2015) (**Table 2**).

Riboflavin concentrations in supernatants of wild-type *C. glutamicum* cultures were low, but traces may be present (**Figure 3**). *Eremothecium ashbyii* and *Ashbya gossypii* are known as natural producers of riboflavin (Osman and Soliman, 1963; Kato and Park, 2011) and *Bacillus subtilis*, *E. coli*, and *Corynebacterium ammoniagenes* were selected and/or metabolically engineered to overproduce riboflavin (Koizumi et al., 2000; Stahmann et al., 2000; Lin et al., 2014). The role of extracellular riboflavin is still unclear. However, iron limitation resulted in riboflavin secretion by *Candida guilliermondii* and other organisms (Enari and Kauppinen, 1961; Neilands, 2014) and it has been suggested that excreted riboflavin may play an important role for ferric iron reduction and iron acquisition (Worst et al., 1998; Crossley et al., 2007). A riboflavin export system is currently unknown.

This study showed that FMN overproduction by *C. glutamicum* is possible. Simultaneous overexpression of *sigH* and *ribF* resulted in the secretion of riboflavin and FMN into the medium, while FAD was not detected (**Figure 4**). Currently, FMN is synthesized chemically involving phosphorylation of riboflavin. However, FMN preparations typically contain ∼25% impurities such as isomeric riboflavin phosphates, riboflavin cyclophosphates, and riboflavin bisphosphates, which can act as antimetabolites and thus be toxic (Abbas and Sibirny, 2011). Enzyme-catalyzed biotransformation of riboflavin and metaphosphate using a crude enzyme preparation from genetically engineered *C. ammoniagenes* yielded 40 μM of FMN without concomitant FAD formation (Nakagawa et al., 1995). Fermentative production of 0.5 mM of FMN using genetically engineered *Candida famata* has also been reported (Yatsyshyn et al., 2010). Although conversion from riboflavin to FMN in the present study was not complete and titers were not high, a proof-of-principle demonstration of fermentative FMN production by *C. glutamicum* could be shown. Future work will address conversion of FMN to FAD and strain development to improve riboflavin, FMN and FAD yields and productivities.

This and work by others (Toyoda et al., 2015) showed that analysis of sigma factor gene overexpression in *C. glutamicum* wild type helped discover the potential of this bacterium for riboflavin production. In *Synechocystis* sp. PCC 6803, overexpression of *sigE* activated expression of sugar catabolic genes and increased polyhydroxybutyrate (PHB) during nitrogen starvation (Osanai et al., 2011, 2013). SigE from *Synechocystis* sp. PCC 6803 and SigB from *C. glutamicum* belong to group 2 sigma factors and SigB from *C. glutamicum* positively regulates glucose catabolism genes (Ehira et al., 2008). Overexpression of SigF in *Mycobacterium smegmatis* enhanced carotenoid biosynthesis by upregulating the carotenoid biosynthesis operon (Kumar et al., 2015), however, *C. glutamicum* does not possess the same type of sigma factor. Future studies will have to establish if and to what extent the approach of sigma factor gene overexpression is transferable to classically obtained or metabolically engineered *C. glutamicum* strains and/or to other bacteria. This may also pertain to “awakening” silent or orphan gene clusters relevant for secondary metabolite production, e.g., silent antibiotic biosynthesis gene clusters.

## Conflict of Interest Statement

The authors declare that the research was conducted in the absence of any commercial or financial relationships that could be construed as a potential conflict of interest.

## References

[B1] Abbas C. A. Sibirny A. A. (2011). Genetic control of biosynthesis and transport of riboflavin and flavin nucleotides and construction of robust biotechnological producers. *Microbiol. Mol. Biol. Rev.* 75 321–360. 10.1128/MMBR.00030-0 21646432 PMC3122625

[B2] Barile M. Brizio C. De Virgilio C. Delfine S. Quagliariello E. Passarella S. (1997). Flavin adenine dinucleotide and flavin mononucleotide metabolism in rat liver–the occurrence of FAD pyrophosphatase and FMN phosphohydrolase in isolated mitochondria. *Eur. J. Biochem.* 249 777–785. 10.1111/j.1432-1033.1997.00777.x 9395326

[B3] Barriuso-Iglesias M. Barreiro C. Sola-Landa A. Martín J. F. (2013). Transcriptional control of the F0F1-ATP synthase operon of *Corynebacterium glutamicum*: sigmaH factor binds to its promoter and regulates its expression at different pH values. *Microb. Biotechnol.* 6 178–188. 10.1111/1751-7915.12022 23298179 PMC3917460

[B4] Blombach B. Arndt A. Auchter M. Eikmanns B. J. (2009). L-Valine production during growth of pyruvate dehydrogenase complex- deficient *Corynebacterium glutamicum* in the presence of ethanol or by inactivation of the transcriptional regulator SugR. *Appl. Environ. Microbiol.* 75 1197–1200. 10.1128/AEM.02351-08 19088318 PMC2643581

[B5] Blombach B. Schreiner M. E. Holátko J. Bartek T. Oldiges M. Eikmanns B. J. (2007). L-valine production with pyruvate dehydrogenase Complex-Deficient *Corynebacterium glutamicum*. *Appl. Environ. Microbiol.* 73 2079–2084. 10.1128/AEM.02826-06 17293513 PMC1855657

[B6] Brune I. Brinkrolf K. Kalinowski J. Pühler A. Tauch A. (2005). The individual and common repertoire of DNA-binding transcriptional regulators of *Corynebacterium glutamicum*, *Corynebacterium efficien*s, *Corynebacterium diphtheriae* and *Corynebacterium jeikeium* deduced from the complete genome sequences. *BMC Genomics* 6:86 10.1186/1471-2164-6-86 PMC1180825 15938759

[B7] Bückle-Vallant V. Krause F. S. Messerschmidt S. Eikmanns B. J. (2014). Metabolic engineering of *Corynebacterium glutamicum* for 2-ketoisocaproate production. *Appl. Microbiol. Biotechnol.* 98 297–311. 10.1007/s00253-013-5310-2 24169948

[B8] Burkovski A. (2008). *Corynebacteria: Genomics and Molecular Biology.* Norfolk, Horizon Scientific Press.

[B9] Busche T. Silar R. Pičmanová M. Pátek M. Kalinowski J. (2012). Transcriptional regulation of the operon encoding stress-responsive ECF sigma factor SigH and its anti-sigma factor RshA, and control of its regulatory network in *Corynebacterium glutamicum*. *BMC Genomics* 13:445 10.1186/1471-2164-13-445 PMC3489674 22943411

[B10] Cho B.-K. Kim D. Knight E. M. Zengler K. Palsson B. O. (2014). Genome-scale reconstruction of the sigma factor network in *Escherichia coli*: topology and functional states. *BMC Biol.* 12:4 10.1186/1741-7007-12-4 PMC3923258 24461193

[B11] Cho S. Yang S. Rhie H. (2012). The gene encoding the alternative thymidylate synthase ThyX is regulated by sigma factor SigB in *Corynebacterium glutamicum* ATCC 13032. *FEMS Microbiol. Lett.* 328 157–165. 10.1111/j.1574-6968.2011.02494.x 22224900

[B12] Crossley R. A. Gaskin D. J. H. Holmes K. Mulholland F. Wells J. M. Kelly D. J. (2007). Riboflavin biosynthesis is associated with assimilatory ferric reduction and iron acquisition by *Campylobacter jejuni*. *Appl. Environ. Microbiol.* 73 7819–7825. 10.1128/AEM.01919-07 17965203 PMC2168145

[B13] Dondrup M. Albaum S. P. Griebel T. Henckel K. Jünemann S. Kahlke T. (2009). EMMA 2-a MAGE-compliant system for the collaborative analysis and integration of microarray data. *BMC Bioinformatics* 10:50 10.1186/1471-2105-10-50 PMC2645365 19200358

[B14] Eberhardt D. Jensen J. V. K. Wendisch V. F. (2014). L-citrulline production by metabolically engineered *Corynebacterium glutamicum* from glucose and alternative carbon sources. *AMB Express* 4:85 10.1186/s13568-014-0085-0 PMC4883986 26267114

[B15] Eggeling L. Bott M. (2005). *Handbook of Corynebacterium glutamicum.* Boca Raton, FL: CRC Press 10.1201/9781420039696

[B16] Eggeling L. Bott M. (2015). A giant market and a powerful metabolism: L-lysine provided by *Corynebacterium glutamicum*. *Appl. Microbiol. Biotechnol.* 99 3387–3394. 10.1007/s00253-015-6508-2. 25761623

[B17] Ehira S. Shirai T. Teramoto H. Inui M. Yukawa H. (2008). Group 2 sigma factor SigB of *Corynebacterium glutamicum* positively regulates glucose metabolism under conditions of oxygen deprivation. *Appl. Environ. Microbiol.* 74 5146–5152. 10.1128/AEM.00944-08 18567683 PMC2519270

[B18] Ehira S. Teramoto H. Inui M. Yukawa H. (2009). Regulation of *Corynebacterium glutamicum* heat shock response by the extracytoplasmic-function sigma factor SigH and transcriptional regulators HspR and HrcA. *J. Bacteriol.* 191 2964–2972. 10.1128/JB.00112-09 19270092 PMC2681815

[B19] Enari T. Kauppinen V. (1961). Interaction of cobalt and iron in riboflavine production of *Candida guilliermondii*. *Acta Chem. Scand.* 15 1513–1516. 10.3891/acta.chem.scand.15-1513

[B20] Feklístov A. Sharon B. D. Darst S. A. Gross C. A. (2014). Bacterial sigma factors: a historical, structural, and genomic perspective. *Annu. Rev. Microbiol.* 68 357–376. 10.1146/annurev-micro-092412-155737 25002089

[B21] Gibson D. G. Young L. Chuang R.-Y. Venter J. C. Hutchison C. A. III Smith H. O. (2009). Enzymatic assembly of DNA molecules up to several hundred kilobases. *Nat. Methods* 6 343–345. 10.1038/nmeth.1318 19363495

[B22] Holátko J. Silar R. Rabatinová A. Sanderová H. Halada P. Nešvera J. (2012). Construction of in vitro transcription system for *Corynebacterium glutamicum* and its use in the recognition of promoters of different classes. *Appl. Microbiol. Biotechnol.* 96 521–529. 10.1007/s00253-012-4336-1 22885668

[B23] Hwang J.-H. Hwang G.-H. Cho J.-Y. (2008). Effect of increased glutamate availability on L-ornithine production in *Corynebacterium glutamicum*. *J. Microbiol. Biotechnol.* 18 704–710. 18467864

[B24] Ikeda M. Baba M. Tsukumoto N. Komatsu T. Mitsuhashi S. Takeno S. (2009). Elucidation of genes relevant to the microaerobic growth of *Corynebacterium glutamicum*. *Biosci. Biotechnol. Biochem.* 73 2806–2808. 10.1271/bbb.90741 19966452

[B25] Ikeda M. Katsumata R. (1999). Hyperproduction of tryptophan by *Corynebacterium glutamicum* with the modified pentose phosphate pathway. *Appl. Environ. Microbiol.* 65 2497–2502. 10347033 10.1128/aem.65.6.2497-2502.1999 PMC91368

[B26] Jensen J. V. K. Wendisch V. F. (2013). Ornithine cyclodeaminase-based proline production by *Corynebacterium glutamicum*. *Microb. Cell Fact.* 12 63 10.1186/1475-2859-12-63 PMC3702523 23806148

[B27] Kalinowski J. Bathe B. Bartels D. Bischoff N. Bott M. Burkovski A. (2003). The complete *Corynebacterium glutamicum* ATCC 13032 genome sequence and its impact on the production of L-aspartate-derived amino acids and vitamins. *J. Biotechnol.* 104 5–25. 10.1016/S0168-1656(03)00154-8 12948626

[B28] Kato T. Park E. Y. (2011). Riboflavin production by *Ashbya gossypii*. *Biotechnol. Lett.* 34 611–618. 10.1007/s10529-011-0833-z 22187081

[B29] Kazmierczak M. J. Wiedmann M. Boor K. J. (2005). Alternative sigma factors and their roles in bacterial virulence. *Microbiol. Mol. Biol. Rev.* 69 527–543. 10.1128/MMBR.69.4.527-543.2005 16339734 PMC1306804

[B30] Kim T.-H. Kim H.-J. Park J.-S. Kim Y. Kim P. Lee H.-S. (2005a). Functional analysis of *sigH* expression in *Corynebacterium glutamicum*. *Biochem. Biophys. Res. Commun.* 331 1542–1547. 10.1016/j.bbrc.2005.04.073 15883048

[B31] Kim T.-H. Park J.-S. Kim H.-J. Kim Y. Kim P. Lee H.-S. (2005b). The *whcE* gene of *Corynebacterium glutamicum* is important for survival following heat and oxidative stress. *Biochem. Biophys. Res. Commun.* 337 757–764. 10.1016/j.bbrc.2005.09.115 16212936

[B32] Kirk-Othmer. (1984). *Encyclopedia of Chemical Technology, Vitamin to Zone Refining* Vol. 24 Edn New York: Wiley-Interscience.

[B33] Koizumi S. Yonetani Y. Maruyama A. Teshiba S. (2000). Production of riboflavin by metabolically engineered *Corynebacterium ammoniagenes*. *Appl. Microbiol. Biotechnol.* 53 674–679. 10.1007/s002539900295 10919325

[B34] Krause F. S. Blombach B. Eikmanns B. J. (2010). Metabolic engineering of *Corynebacterium glutamicum* for 2-ketoisovalerate production. *Appl. Environ. Microbiol.* 76 8053–8061. 10.1128/AEM.01710-10 20935122 PMC3008247

[B35] Kumar S. Matange N. Umapathy S. Visweswariah S. S. (2015). Linking carbon metabolism to carotenoid production in mycobacteria using Raman spectroscopy. *FEMS Microbiol. Lett.* 362 1–6. 10.1093/femsle/fnu048 25673658

[B36] Larisch C. Nakunst D. Hüser A. T. Tauch A. Kalinowski J. (2007). The alternative sigma factor SigB of *Corynebacterium glutamicum* modulates global gene expression during transition from exponential growth to stationary phase. *BMC Genomics* 8:4 10.1186/1471-2164-8-4 PMC1779776 17204139

[B37] Lin Z. Xu Z. Li Y. Wang Z. Chen T. Zhao X. (2014). Metabolic engineering of *Escherichia coli* for the production of riboflavin. *Microb. Cell Fact.* 13:104 10.1186/s12934-014-0104-5 PMC4223517 25027702

[B38] Mimitsuka T. Sawai H. Hatsu M. Yamada K. (2007). Metabolic engineering of *Corynebacterium glutamicum* for cadaverine fermentation. *Biosci. Biotechnol. Biochem.* 71 2130–2135. 10.1271/bbb.60699 17895539

[B39] Morbach S. Sahm H. Eggeling L. (1996). L-Isoleucine production with *Corynebacterium glutamicum*: further flux increase and limitation of export. *Appl. Environ. Microbiol.* 62 4345–4351. 16535457 10.1128/aem.62.12.4345-4351.1996 PMC1388995

[B40] Nakagawa S. Hagihara T. Fujio T. Aisaka K. (1995). Metaphosphate-dependent phosphorylation of riboflavin to FMN by *Corynebacterium ammoniagenes*. *Appl. Microbiol. Biotechnol.* 43 325–329. 10.1007/BF00172833

[B41] Nakunst D. Larisch C. Hüser A. T. Tauch A. Pühler A. Kalinowski J. (2007). The extracytoplasmic function-type sigma factor SigM of *Corynebacterium glutamicum* ATCC 13032 is involved in transcription of disulfide stress-related genes. *J. Bacteriol.* 189 4696–4707. 10.1128/JB.00382-07 17483229 PMC1913457

[B42] Neilands J. B. (2014). *Microbial Iron Metabolism: A Comprehensive Treatise.* New York: Academic Press.

[B43] Netzer R. Krause M. Rittmann D. Peters-Wendisch P. G. Eggeling L. Wendisch V. F. (2004). Roles of pyruvate kinase and malic enzyme in *Corynebacterium glutamicum* for growth on carbon sources requiring gluconeogenesis. *Arch. Microbiol.* 182 354–363. 10.1007/s00203-004-0710-4 15375646

[B44] Nováková J. Izsáková A. Grivalský T. Ottmann C. Farkašovský M. (2014). Improved method for high-efficiency electrotransformation of *Escherichia coli* with the large BAC plasmids. *Folia Microbiol. (Praha)* 59 53–61. 10.1007/s12223-013-0267-1 23846555

[B45] Osanai T. Numata K. Oikawa A. Kuwahara A. Iijima H. Doi Y. (2013). Increased bioplastic production with an RNA polymerase sigma factor SigE during nitrogen starvation in *Synechocystis* sp. PCC 6803. *DNA Res.* 20 525–535. 10.1093/dnares/dst028 23861321 PMC3859321

[B46] Osanai T. Oikawa A. Azuma M. Tanaka K. Saito K. Hirai M. Y. (2011). Genetic engineering of group 2 sigma factor SigE widely activates expressions of sugar catabolic genes in *Synechocystis* species PCC 6803. *J. Biol. Chem.* 286 30962–30971. 10.1074/jbc.M111.231183 21757761 PMC3162455

[B47] Osman H. G. Soliman M. H. M. (1963). Biosynthesis of riboflavin (Vit. B2) by *Eremothecium ashbyii*. *Arch. Mikrobiol.* 46 255–264. 10.1007/BF00422187 14098802

[B48] Paget M. S. Helmann J. D. (2003). The σ70 family of sigma factors. *Genome Biol.* 4:203 10.1186/gb-2003-4-1-203 PMC151288 12540296

[B49] Park S.-D. Youn J.-W. Kim Y.-J. Lee S.-M. Kim Y. Lee H.-S. (2008). *Corynebacterium glutamicum* sigmaE is involved in responses to cell surface stresses and its activity is controlled by the anti-sigma factor CseE. *Microbiol. Read. Engl.* 154 915–923. 10.1099/mic.0.2007/012690-0 18310037

[B50] Park S. H. Kim H. U. Kim T. Y. Park J. S. Kim S.-S. Lee S. Y. (2014). Metabolic engineering of *Corynebacterium glutamicum* for L-arginine production. *Nat. Commun.* 5:4618 10.1038/ncomms5618 25091334

[B51] Pátek M. Nešvera J. (2011). Sigma factors and promoters in *Corynebacterium glutamicum*. *J. Biotechnol.* 154 101–113. 10.1016/j.jbiotec.2011.01.017 21277915

[B52] Peters-Wendisch P. G. Schiel B. Wendisch V. F. Katsoulidis E. Möckel B. Sahm H. (2001). Pyruvate carboxylase is a major bottleneck for glutamate and lysine production by *Corynebacterium glutamicum*. *J. Mol. Microbiol. Biotechnol.* 3 295–300. 11321586

[B53] Peters-Wendisch P. Stolz M. Etterich H. Kennerknecht N. Sahm H. Eggeling L. (2005). Metabolic engineering of *Corynebacterium glutamicum* for L-serine production. *Appl. Environ. Microbiol.* 71 7139–7144. 10.1128/AEM.71.11.7139-7144.2005 16269752 PMC1287687

[B54] Pfeifer-Sancar K. Mentz A. Rückert C. Kalinowski J. (2013). Comprehensive analysis of the *Corynebacterium glutamicum* transcriptome using an improved RNAseq technique. *BMC Genomics* 14:888 10.1186/1471-2164-14-888 PMC3890552 24341750

[B55] Polen T. Schluesener D. Poetsch A. Bott M. Wendisch V. F. (2007). Characterization of citrate utilization in *Corynebacterium glutamicum* by transcriptome and proteome analysis. *FEMS Microbiol. Lett.* 273 109–119. 10.1111/j.1574-6968.2007.00793.x 17559405

[B56] Radmacher E. Vaitsikova A. Burger U. Krumbach K. Sahm H. Eggeling L. (2002). Linking central metabolism with increased pathway flux: L-valine accumulation by *Corynebacterium glutamicum*. *Appl. Environ. Microbiol.* 68 2246–2250. 10.1128/AEM.68.5.2246-2250.2002 11976094 PMC127577

[B57] Sambrook J. (2001). *Molecular Cloning: A Laboratory Manual, 3rd Edn*. Cold Spring Harbor, NY: Cold Spring Harbor Laboratory Press.

[B58] Schneider J. Eberhardt D. Wendisch V. F. (2012). Improving putrescine production by *Corynebacterium glutamicum* by fine-tuning ornithine transcarbamoylase activity using a plasmid addiction system. *Appl. Microbiol. Biotechnol.* 95 169–178. 10.1007/s00253-012-3956-9 22370950

[B59] Schneider J. Wendisch V. F. (2010). Putrescine production by engineered *Corynebacterium glutamicum*. *Appl. Microbiol. Biotechnol.* 88 859–868. 10.1007/s00253-010-2778-x 20661733

[B60] Sharma U. K. Chatterji D. (2010). Transcriptional switching in *Escherichia coli* during stress and starvation by modulation of σ70 activity. *FEMS Microbiol. Rev.* 34 646–657. 10.1111/j.1574-6976.2010.00223.x 20491934

[B61] Si M. Long M. Chaudhry M. T. Xu Y. Zhang P. Zhang L. (2014). Functional characterization of *Corynebacterium glutamicum* mycothiol S-conjugate amidase. *PLoS ONE* 9:e115075 10.1371/journal.pone.0115075 PMC4267739 25514023

[B62] Si M. Xu Y. Wang T. Long M. Ding W. Chen C. (2015a). Functional characterization of a mycothiol peroxidase in *Corynebacterium glutamicum* that uses both mycoredoxin and thioredoxin reducing systems as proton donor for oxidative stress response. *Biochem. J.* 10.1042/BJ20141080 [Epub ahead of print]. 25891483

[B63] Si M. Zhang L. Chaudhry M. T. Ding W. Xu Y. Chen C. (2015b). *Corynebacterium glutamicum* methionine sulfoxide reductase A uses both mycoredoxin and thioredoxin for regeneration and oxidative stress resistance. *Appl. Environ. Microbiol.* 81 2781–2796. 10.1128/AEM.04221-14. 25681179 PMC4375309

[B64] Stahmann K. P. Revuelta J. L. Seulberger H. (2000). Three biotechnical processes using Ashbya gossypii, Candida famata, or Bacillus subtilis compete with chemical riboflavin production. *Appl. Microbiol. Biotechnol.* 53 509–516. 10.1007/s002530051649 10855708

[B65] Stansen C. Uy D. Delaunay S. Eggeling L. Goergen J.-L. Wendisch V. F. (2005). Characterization of a *Corynebacterium glutamicum* lactate utilization operon induced during temperature-triggered glutamate production. *Appl. Environ. Microbiol.* 71 5920–5928. 10.1128/AEM.71.10.5920-5928.2005 16204505 PMC1265975

[B66] Staroń A. Sofia H. J. Dietrich S. Ulrich L. E. Liesegang H. Mascher T. (2009). The third pillar of bacterial signal transduction: classification of the extracytoplasmic function (ECF) σ factor protein family. *Mol. Microbiol.* 74 557–581. 10.1111/j.1365-2958.2009.06870.x 19737356

[B67] Takemoto N. Tanaka Y. Inui M. (2015). Rho and RNase play a central role in FMN riboswitch regulation in *Corynebacterium glutamicum*. *Nucleic Acids Res.* 43 520–529. 10.1093/nar/gku1281 25477389 PMC4288175

[B68] Takemoto N. Tanaka Y. Inui M. Yukawa H. (2014). The physiological role of riboflavin transporter and involvement of FMN-riboswitch in its gene expression in *Corynebacterium glutamicum*. *Appl. Microbiol. Biotechnol.* 98 4159–4168. 10.1007/s00253-014-5570-5 24531272

[B69] Toyoda K. Teramoto H. Yukawa H. Inui M. (2015). Expanding the regulatory network governed by the extracytoplasmic function sigma factor σH in *Corynebacterium glutamicum*. *J. Bacteriol.* 197 483–496. 10.1128/JB.02248-14 25404703 PMC4285989

[B70] Tripathi L. Zhang Y. Lin Z. (2014). Bacterial sigma factors as targets for engineered or synthetic transcriptional control. *Front. Bioeng. Biotechnol.* 2:33 10.3389/fbioe.2014.00033 PMC4153023 25232540

[B71] van der Rest M. E. Lange C. Molenaar D. (1999). A heat shock following electroporation induces highly efficient transformation of *Corynebacterium glutamicum* with xenogeneic plasmid DNA. *Appl. Microbiol. Biotechnol.* 52 541–545. 10.1007/s002530051557 10570802

[B72] Vassylyev D. G. Sekine S. Laptenko O. Lee J. Vassylyeva M. N. Borukhov S. (2002). Crystal structure of a bacterial RNA polymerase holoenzyme at 2.6 A resolution. *Nature* 417 712–719. 10.1038/nature752 12000971

[B73] Vogl C. Grill S. Schilling O. Stülke J. Mack M. Stolz J. (2007). Characterization of riboflavin (Vitamin B2) transport proteins from Bacillus subtilis and *Corynebacterium glutamicum*. *J. Bacteriol.* 189 7367–7375. 10.1128/JB.00590-07 17693491 PMC2168442

[B74] Vogt M. Haas S. Polen T. van Ooyen J. Bott M. (2015). Production of 2-ketoisocaproate with *Corynebacterium glutamicum* strains devoid of plasmids and heterologous genes. *Microb. Biotechnol.* 8 351–360. 10.1111/1751-7915.12237 25488800 PMC4353348

[B75] Watanabe K. Teramoto H. Suzuki N. Inui M. Yukawa H. (2013). Influence of SigB inactivation on *Corynebacterium glutamicum* protein secretion. *Appl. Microbiol. Biotechnol.* 97 4917–4926. 10.1007/s00253-012-4586-y 23179627

[B76] Weber H. Polen T. Heuveling J. Wendisch V. F. Hengge R. (2005). Genome-wide analysis of the general stress response network in *Escherichia coli*: σS-dependent cenes, promoters, and sigma factor selectivity. *J. Bacteriol.* 187 1591–1603. 10.1128/JB.187.5.1591-1603.2005 15716429 PMC1063999

[B77] Wendisch V. F. (2003). Genome-wide expression analysis in *Corynebacterium glutamicum* using DNA microarrays. *J. Biotechnol.* 104 273–285. 10.1016/S0168-1656(03)00147-0 12948645

[B78] Worst D. J. M. Gerrits M. Vandenbroucke-Grauls C. M. J. E. Kusters J. G. (1998). *Helicobacter pylori ribBA*-mediated riboflavin production is involved in iron acquisition. *J. Bacteriol.* 180 1473–1479. 9515916 10.1128/jb.180.6.1473-1479.1998 PMC107047

[B79] Yatsyshyn V. Y. Fedorovych D. V. Sibirny A. A. (2010). Medium optimization for production of flavin mononucleotide by the recombinant strain of the yeast *Candida famata* using statistical designs. *Biochem. Eng. J.* 49 52–60. 10.1016/j.bej.2009.11.010

[B80] Yukawa H. Inui M. (eds) (2013). *Corynebacterium glutamicum.* Berlin: Springer Berlin 10.1007/978-3-642-29857-8

[B81] Zemanová M. Kaderábková P. Pátek M. Knoppová M. Silar R. Nesvera J. (2008). Chromosomally encoded small antisense RNA in *Corynebacterium glutamicum*. *FEMS Microbiol. Lett.* 279 195–201. 10.1111/j.1574-6968.2007.01024.x 18093135

